# A Review of FDA-Approved Multi-Target Angiogenesis Drugs for Brain Tumor Therapy

**DOI:** 10.3390/ijms26052192

**Published:** 2025-02-28

**Authors:** Iuliana Mihaela Buzatu, Ligia Gabriela Tataranu, Carmen Duta, Irina Stoian, Oana Alexandru, Anica Dricu

**Affiliations:** 1 Department of Microbiology, “Fundeni” Clinical Institute, Șoseaua Fundeni 258, 022328 Bucharest, Romania; buzatu_iuliana@yahoo.com; 2 Department of Neurosurgery, Clinical Emergency Hospital “Bagdasar-Arseni”, Soseaua Berceni 12, 041915 Bucharest, Romania; ligia.tataranu@umfcd.ro; 3 Department of Neurosurgery, Carol Davila University of Medicine and Pharmacy, 020021 Bucharest, Romania; 4 Department of Biochemistry, Carol Davila University of Medicine and Pharmacy, 020022 Bucharest, Romania; carmen.duta@umfcd.ro (C.D.); irina.stoian@umfcd.ro (I.S.); anica.dricu@umfcd.ro (A.D.); 5 Department of Neurology, University of Medicine and Pharmacy of Craiova, Petru Rares 2, 200349 Craiova, Romania

**Keywords:** glioblastoma, angiogenesis, tyrosine kinase inhibitors, multi-target therapy, brain tumors

## Abstract

Neovascularization is an important process in brain tumor development, invasion and metastasis. Several research studies have indicated that the VEGF signaling target has potential for reducing angiogenesis in brain tumors. However, targeting VEGF signaling has not met the expected efficacy, despite initial enthusiasm. This is partly because tumors cleverly use alternative growth factor pathways, other than VEGF signaling, to restore angiogenesis. Multi-target inhibitors have been developed to inhibit several receptor kinases that play a role in the development of angiogenesis. By simultaneously affecting various receptor kinases, these treatments can potentially obstruct various angiogenic pathways that are involved in brain cancer advancement, often offering a more holistic strategy than treatments focusing on just one kinase. Since 2009, the FDA has approved a number of multi-kinase inhibitors that target angiogenic growth factor receptors (e.g., VEGFR, PDGFR, FGFR, RET, c-KIT, MET, AXL and others) for treatment of malignant diseases, including brain cancer. Here, we present some recent results from the literature regarding the preclinical and clinical effects of these inhibitors on brain tumors.

## 1. Introduction

In brain tumors, a favorable microenvironment, characterized by hypoxia and extensive growth factor secretion, frequently induces a broad neovascularization, which in turn makes the tumor more forceful and resistant to treatment like radiation or chemotherapy. Angiogenesis is known as a hallmark of cancers; it has also been demonstrated that the glial tumors develop new capillary blood vessels capable of supporting the tumor’s growth [1]. This is a result of the imbalance between the pro-angiogenic and anti-angiogenic regulators [2]. Among the pro-angiogenic growth factors are vascular endothelial growth factor (VEGF), platelet-derived growth factor (PDGF), basic fibroblast growth factor (bFGF) and angiopoetin (Ang). Angiostatin and endostatin are the anti-angiogenic factors [3].

The receptors of the pro-angiogenic growth factors are vascular-endothelial growth factor receptors (VEGFRs), platelet-derived growth factor receptors (PDGFRs), fibroblast growth factor receptors (FGFRs) and Tie receptors. The binding of VEGF, PDGF and bFGF to the cognate receptors drives dimerization and stimulates the activation of several intracellular signaling pathways. For instance, VEGFR may induce the activation of the Ras/Raf/MEK/ERK pathway or the phospholipase-Cγ/protein kinase C(PLCy/PKC) pathway. These pathways are capable of regulating endothelial cell proliferation and migration, but also vascular permeability [4]. The phosphatidylinositol-3 kinase (PI3K)/phosphatase and tensin homologue (PTEN)/Akt/mammalian target of rapamycin (mTOR) is another important signaling pathway which is involved in vascular permeability, but also in endothelial cell survival. It may be activated by PDGFR, but also by PDGFR or bFGFR [5,6,7]. Tie-2 stimulates some signaling pathways through the Tie-2 receptor, also known as TEK, Ang-1. Some of these pathways are common with those activated by the dimerization of other pro-angiogenic growth factors such as Ras/Raf/MEK.ERK or PI3K/Akt/mTOR [8].

Brain tumor growth requests oxygen and nutrition, both supplied by new blood vessels. This process is triggered by hypoxia, which induces the expression of hypoxia-inducible factor-1 (Hif-1). Hif-1 is capable of activating the transcription of pro-angiogenic growth factors, including VEGF [9]. VEGF expression is correlated with cerebral microvascular proliferation: almost absent in low-grade gliomas and highly expressed in high-grade gliomas [10]. Instead, meningiomas, although highly vascularized, are less aggressive [11]. Both bFGF and PDGF are involved in recruiting peri-endothelial cells to vessels and are important pro-angiogenic regulators in brain tumors [12,13]. What is interesting is that physiological brain angiogenesis is regulated by a similar mechanism. The equilibrium between the pro-angiogenic and the anti-angiogenic factors plays a pivotal role in brain angiogenesis. During embryogenesis, VEGF expression is augmented in the neuroectoderm, while, in adult life, brain angiogenesis is absent and VEGF expression is at very low range. In highly malignant brain tumors, the VEGF level increases gradually to reach values similar to those initially found in embryogenesis [14,15,16].

**Figure 1:**
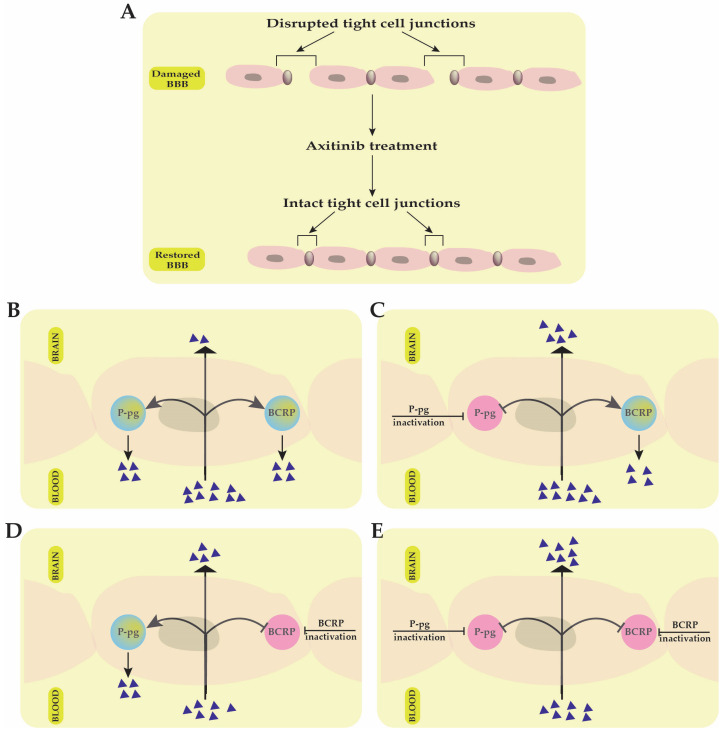
The effectiveness of multi-target kinase inhibitors in treating brain tumors is significantly influenced by their ability to cross the blood–brain barrier (BBB). (**A**) In malignant brain tumors, the BBB structure can be disrupted due to the loss of tight junction proteins, increasing permeability. Treatment with axitinib helps normalize this barrier by restoring the integrity of tight cell junctions. (**B**) ATP-binding cassette (ABC) transporters, such as P-glycoprotein (P-gp) and Breast Cancer Resistance Protein (BCRP), limit the penetration of multi-target kinase inhibitors into the brain by actively transporting them back into the bloodstream. (**C**) Inhibiting P-gp improves drug penetration into the brain, but BCRP continues to restrict drug transport into brain tissue. (**D**) Inhibiting BCRP increases intracerebral drug accumulation, but P-gp still reduces the efficiency of BBB crossing. (**E**) Simultaneous inhibition of both P-gp and BCRP allows improved penetration of multi-target kinase inhibitors, thereby enhancing the effectiveness of tumor treatment.

Multi-target drug effectiveness in brain tumors is significantly influenced by the drug’s capacity to pass the brain–blood barrier (BBB). The BBB is mainly made up of endothelial cells, which form tight junctions that restrict anticancer drugs’ transport to the brain (Figure 1). Multi-target kinase inhibitors (i.e., axitinib, sorafenib, lenvatinib, pazopanib, sunitinib, cabozantinib, nintedanib and regorafenib) are relatively large, lipophilic molecules, and as a result, they have limited passive diffusion through the tight endothelial cell junctions that form the BBB. This limits their efficacy in treating brain tumors, particularly primary brain cancers or brain metastases. In malignant diseases, like brain cancer, several sites of the BBB may be disrupted by the loss of tight junction proteins, resulting in increased permeability (Figure 1A) [17]. The multi-kinase inhibitor axitinib was reported to assist BBB normalization/permeabilization in brain tumors, thus improving brain tumor response to treatment [18,19] (Figure 1A).

Interestingly, in a recent study by Kai Wang et al., published in 2024, axitinib was also found to have a protective effect on BBB alteration and cerebral ischemia-induced damage by attenuating the tight junction proteins’ injury [20].

The ATP-binding cassette efflux transporters further complicate BBB drug penetration in cancer. Two major proteins have also been shown to restrict drug transport across the BBB into the brain: ATP-binding cassette transporters P-glycoprotein (P-gp) and breast cancer resistance protein (BCRP) (Figure 1). Several kinase inhibitors have been suggested to be substrates for these two multi-drug efflux transporters, further complicating their ability to traverse the BBB (Figure 1B) [21,22]. For example, in GB, the cooperation between the P-gp and BCRP transporters was demonstrated to restrict the ability of sunitinib to efficiently bypass the BBB, while inactivation of these efflux protein transporters improved the brain delivery and treatment efficacy of the drug (Figure 1C–E) [23].

Among the angiogenic inhibitors mentioned above, axitinib and sunitinib have been the best studied in terms of interaction with the BBB in cancer. Concerning brain tumors, several FDA-approved multi-target angiogenesis inhibitors (e.g., sorafenib, lenvatinib, pazopanib, cabozantinib, nintedanib, regorafenib) have undergone clinical trials and their ability to cross the BBB may be very different depending on the molecular weight, lipophilicity, bioavailability or systemic concentration of the drug. Unfortunately, there are no studies available so far that can clearly describe the BBB crossing mechanism for these angiogenic inhibitors.

Vasogenic oedema is another cause of morbidity in brain tumor patients. The same heterogenicity is encountered in medulloblastomas with different types of fenestration of the endothelial cells [24]. The consequence of this heterogenicity is the unequal distribution of drugs into brain tumors.

The hypothesis that angiogenesis was extremely important for the growth of brain tumors raised hopes for the therapeutic potential of anti-angiogenic therapies. Given the significance of the VEGF pathway in the development of cancer angiogenesis and the prevalence of VEGF in brain tumors, brain cancer therapies were nearly solely focused on blocking the VEGF pathway [25,26]. As a result, in May 2009, the Food and Drug Administration (FDA) approved the use of bevacizumab (Avastin, Genentech, Inc., South San Francisco, CA, USA) as a single agent for GBM patients with progressive disease following prior therapy [27]. Bevacizumab is a recombinant humanized monoclonal antibody, which targets all VEGF isoforms. The drug was tested either alone or in combination with other treatment modalities in GBM clinical trials. Although the single or combined regimen exceeded anterior records, the results were rather disappointing: the drug prolonged progression-free survival (PFS), and produced an improvement in neurological signs and a reduction in steroid intake, but did not improve overall survival (OS) [28,29,30,31]. In meningiomas, VEGF is also largely expressed, and the expression increases with meningioma grade. In this light, bevacizumab has shown some positive results. However, the prospective trials were rather small and there was a lack of control [32]. Other monoclonal antibodies that have been studied, either in preclinical or clinical studies, are tanibirumab or MSB0254. These drugs need further testing [33,34]. Our previous study showed that SU1498, a VEGFR inhibitor, had a cytotoxic effect on high-grade glioma cells (HGG), but the effect was rather limited [35]. Therapy resistance may be a cause of the limited response to treatment. For instance, there are patients diagnosed with GBM who do not respond to bevacizumab therapy because the tumor has intrinsic resistance to anti-angiogenic therapy. On the other hand, there are patients who have an initial response to Avastin treatment but may develop further resistance due to upregulation of other pro-angiogenic pathways, increased pericity coverage or increased invasiveness of tumor cells that determines the capacity to co-opt pre-existing brain blood vessels [36]. Some scientists have also discussed the role of biomarkers, which can be associated with tumor progression and angiogenesis but also with resistance to therapy [37,38,39,40].

Because angiogenesis is regulated by the crosstalk between multiple signaling molecules and various signaling pathways, dogma claims that multi-target drugs can potentially obstruct various angiogenic pathways. Single-target therapy generally targets proteins or genes with the purpose of stopping malignant cell survival and proliferation. Besides the positive effects, monotherapy is characterized by a lack of selectivity towards healthy cells. Another difficulty is the development of tumor cell resistance to single-target therapies [41]. On the other hand, the combination of drugs which have different mechanisms of action in the end, can lead to synergistic antitumor effects. This therapeutical possibility is also accompanied by negative effects like the summation of side effects of each drug, the high cost, or sometimes the side effects provoked by the interaction between drugs [42]. Multi-drug therapies have an important characteristic: one compound has high affinity to multiple targets at the same time. This is a way to reduce toxicity, the side effects, or costs, and, at the same time, to increase the efficacy of cancer treatment [43]. The purpose of anti-angiogenic therapy is the normalization of tumoral blood vessels, reduction or loss of hypoxia and the limitation of tumor invasion and metastasis, but also the improvement of the drug concentration in the tumor tissue [44]. For instance, bevacizumab, the first anti-angiogenic drug approved by the FDA, achieved some positive results either alone or in combination with other drugs or radiation therapy. However, the benefits of bevacizumab treatment did not meet expectations. Moreover, the FDA decided to withdraw bevacizumab for treatment of HER-2-negative breast cancer, in which the drug was not shown to be safe and efficient [45]. There are also other types of tumors where bevacizumab failed to demonstrate a significant anticancer effect: pancreatic cancer, gastric cancer and prostate cancer. One explanation could be the interference between the angiogenic signaling pathway with other signaling pathways [46].

This hypothesis led to the idea of developing multi-targeted anti-angiogenic therapies capable of overcoming resistance to anti-angiogenic monotherapy. Investigators embraced the idea of designing new, more potent and efficient anticancer drugs with multi-target properties [47]. Some of these treatments already have a success story. For instance, multi-target tyrosine kinases inhibitors like axitinib, sorafenib, lenvatinib, pazopanib, sunitinib, cabozantinib, nintedanib or regorafenib have already received FDA approval for the treatment of various solid tumors. Advanced stages of cancers like renal cell cancer, thyroid cancer, hepatocellular carcinoma, metastatic colorectal cancer, gastrointestinal stromal tumors and non-small cell lung cancer have seen improvements in survival due to this anti-angiogenic therapy. Similar results have been obtained by using multi-target monoclonal antibodies like aflibercept or ramucirumab for metastatic colorectal cancer, gastric cancer or non-small cell lung cancer [48]. Regarding brain tumors, these types of therapy are still under investigation.

Here, we focus on presenting the data regarding the efficacy of FDA-approved multi-target, anti-angiogenic drugs for brain tumor therapy. We will discuss preclinical in vitro and in vivo studies, but also the key clinical investigations.

## 2. Kinases as Targets for Anti-Angiogenic Therapy

VEGFR is among the tyrosine-kinases (TKRs) that are overexpressed in the endothelial cells of GBM. VEGF signaling is mediated through VEGFRs like VEGFR-1 (Flt-1), VEGFR-2 (KDR or FLK-1) or VEGFR-3 (FLT-4). These receptors have a transmembrane domain, an extracellular ligand-binding domain and a tyrosine kinase with an intracellular domain [49]. VEGFR-1 plays an important role in tumor angiogenesis and it is overexpressed in GBM cells [50], while in EGFRvIII-positive glioblastoma cells it has been observed that VEGFR-2 is mainly overexpressed [51]. It has also been demonstrated that, in GBM cells, a high level of VEGFR-3 is present [52]. The receptor, mainly VEGFR-2, is also overexpressed in meningiomas. In fact, VEGFR2 expression is significantly correlated with the WHO grade of the tumor [53].

Regarding the inhibition of angiogenesis in brain tumor patients since 2009, the US FDA has approved the inhibition of the VEGF-A/VEGFR-2 axis with bevacizumab for recurrent GBM. Since 2021, the drug has been included in European Association of Neuro-Oncology (EANO) guidelines due to its demonstrated improvement in quality of life and safety [54]. The drug has also been tested in meningioma patients but further clinical trials are needed [32].

The PDGF growth factors bind and activate two RTKs: PDGFR-α and PDGFR-β. This leads to receptor dimerization, transphosphorylation and activation of intracellular signaling pathways. The receptors have a transmembrane domain, a juxta-membrane domain, a kinase insertion domain, an intracellular domain and five extracellular Ig-like domains [55]. In normal conditions, they are important players involved in angiogenesis, growth and reproduction of endothelial cells, vascular maturation, revascularization and wound healing [56]. However, PDGFRs are expressed in a range of malignant tumor cells [57]. For instance, the amplification of PDGFR-α was found in GBM [58,59] and was associated with poor overall survival [60]. The overexpression of PDGFR-α in association with p53 mutation in GBM patients proved to be the least responsive to treatment [61]. A crosstalk between PDGF/PDGFR and other signaling pathways was also observed. An example of this is the PDGFRα- phosphatidylinositol 3-kinase-protein kinase B (PI3K-AKT) signaling pathway, which is activated in 70% of GBM [62]. The tumor invasion and angiogenesis promoted by AKT activation is a consequence of the overexpression of PDGFR-α induced by hypoxia [63].

PDGF-B and PDGFR-β are also overexpressed in GBM [64,65,66]. They are responsible for vessel maturation through the recruitment of peri-endothelial cells to vessels. PDGF-B stimulates glioma angiogenesis by inducing VEGF expression in tumor endothelial and perycite recruitment to growing vessels [67]. In fact, in astrocytoma grades I, II and III, angiogenesis is almost absent, while in GBM there is increased expression of PDGF-B and PDGFR-β. This event provokes the upregulation of VEGF in the tumor endothelium. In the end, all these processes determine the recruitment of pericytes [13]. Therefore, PDGF-B and PDGFR-β stimulate GBM angiogenesis. Meningiomas are also highly vascular tumors and most meningiomas express PDGFRβ [68].

The cognate receptors of FGFs are the four fibroblast growth factor receptors (FGFRs) 1 to 4. These receptors contain three extracellular immunoglobulin-like binding domains, a transmembrane domain and an intracellular domain which constitutes a two-part tyrosine kinase [69]. The binding of FGFs to FGFRs determines receptor dimerization and leads to autophosphorylation of tyrosine residues. The result is the activation of multiple signaling pathways like PI-3/AKT/mammalian target of rapamycin(mTOR) or the STAT3/NF-κB signaling pathway [70]. FGF2 is a growth factor involved in GBM vascularization [71]. The FGF2/FGFRs system is potentially an important target to block glioma angiogenesis [72]. FGF is also involved in meningioma angiogenesis [73].

The ANG family of growth factors has two receptor tyrosine kinases: Tie 1 and Tie2, also known as TEK [74]. The Tie 2 receptors are expressed by the endothelial cells. It has been observed that ANG1 is produced by pericytes and smooth muscle cells. This glycoprotein binds to the Tie 2 receptor and is very important in the process of recruitment of perycites and smooth muscle cells, but also in the remodeling of the vessel wall or for endothelial sprouting [75]. The other glycoprotein, ANG2, is produced by the endothelial cells. It also binds to Tie 2 receptor tyrosine kinase but does not activate the receptor. It is an antagonistic factor [76]. Unlike ANG1 and ANG2, ANGPTL4 does not bind to the ANG receptors Tie 1 and Tie 2 to mediate their biological functions. Its cognate receptors are unknown. However, it has been detected in a variety of organs or tissues like the liver, adipose tissues, kidneys, skin and intestines [77]. There are studies that emphasize the role of ANGPTL4 in tumor angiogenesis [78]. In 2010, Brunckhorst et al. demonstrated that ANGPTL4 promoted tumor angiogenesis in human GBM cells [79]. Also, ANG-1 and ANG-2 are expressed in GBM tumor cells and vessels [80]. The concomitant targeting of ANG-2 (with trebananib) and VEGF (with bevacizumab) improved vascular normalization and survival in GBM [81]. It has also been demonstrated that the ANG-2 plasma concentration of patients with meningiomas is rather high [82].

## 3. Evaluation of FDA-Approved Multi-Target Anti-Angiogenic Inhibitors in Preclinical Models of Brain Tumors

### 3.1. Axitinib

**Figure 2:**
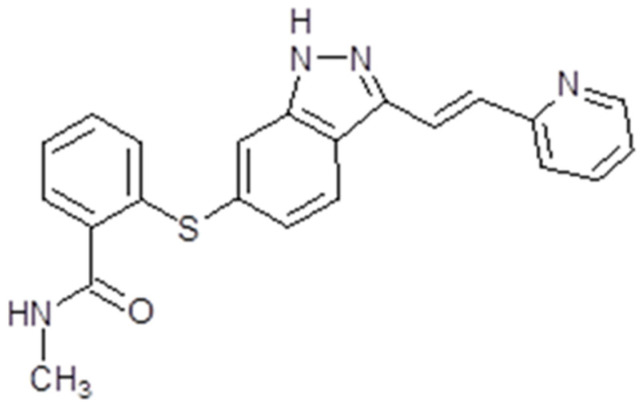
Axitinib chemical structure. The design was created with ACD/ChemSketch 2.0 (Freeware).

**Figure 3:**
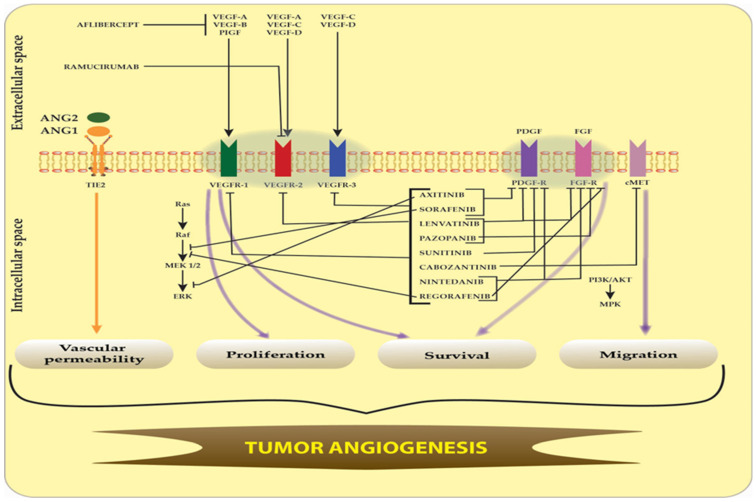
Targeted inhibition pathways of VEGFR, PDGFR, FGFR, cMET and TIE-2 receptors by multi-kinase inhibitors. The blockade of VEGFR, PDGFR, FGFR, cMET and TIE-2 receptors and their signaling pathways by multi-kinase inhibitors leads to inhibition of glioma vascular permeability, migration, proliferation and survival within angiogenesis. The main FDA-approved multi-target angiogenesis drugs are axitinib, sorafenib, lenvatinib, pazopanib, sunitinib, cabozantinib, nintedanib, regorafenib, aflibercept and ramucirumab. Sharp arrows (→) illustrate stimulation while blunt arrows (┴) indicate inhibition.

Axitinib, also known as AG013736 or Inlya (Pfizer Inc., New York, NY, USA) is a TK inhibitor [83]. This synthetic drug is an indazole derivative with a molecular weight of 386.47 Da (Figure 2) [84]. As a TK inhibitor, axitinib is capable of binding to the catalytic domain of VEGFRs. In fact, the drug is a potent inhibitor of VEGFR 1, 2 and 3 activities [85]. Regarding the mechanism of action, at picomolar concentration, it is capable of binding the intracellular ATP site of VEGFR in a competitive manner. The final result is the inhibition of signal transduction by VEGF. Axitinib also acts on the endothelial cells. Here, by blocking the VEGF/VEGFR pathways, it has the result of diminishing the phosphorylation of some protein kinases like AKT, or mitogen-activated protein kinases (ERK1/2). The phosphorylation of nitric oxide synthase (eNOS) is also affected. As a multi-target TK inhibitor, it can also suppress PDGFR and EGFR 1, 2 and 3, but also the gene cKIT. This is possible when the drug is administrated in picomolar concentrations [86] (Figure 3).

The pharmacological presentation is tablets. After oral intake, the maximum plasma concentration occurs in about 4 h; therefore, the absorption process is rapid. Its metabolism is mainly in the liver, while the excretion is hepatobiliary [87]. Being metabolized in the liver, the pharmacokinetics are influenced by cytocrome P450 inducers like, for instance, phenytoin, or by CYP inhibitors like ketoconazole. It has a 58% oral bioavailability. Therefore, a dose reduction is needed in patients with hepatic diseases. The clearance of axitinib is not affected by the renal impairment [88].

Axitinib was tested in preclinical studies for different types of tumors: thyroid cancer [89], epithelial ovarian cancer [90], nasopharyngeal cancer [91], hepatocellular carcinoma (HCC) [92], non-small cell lung cancer [93], metastatic melanoma [94], pancreatic cancer, breast cancer [95] and colorectal cancer [96].

For the treatment of brain tumors, axitinib still had not been studied enough. In 2014, Wakimoto et al. demonstrated a direct cytotoxic effect against a number of patient-derived GBM stem cells and an endothelial cell line. The authors also proved that the drug has the capacity to prolong survival and decreased the tumor-associated vascularity in orthotopic GBM models [97]. In 2016, the capacity of axitinib to induce senescence-associated cell death and necrosis in glioma cell lines was demonstrated by Santoni et al. In the same study, the cytotoxic effect of the drug was demonstrated in combination with bortezomib mainly [98]. The drug is also capable of diminishing the proliferation and motility of human GBM cells [99]. In another in vitro study, axitinib retarded cell growth and had a cytotoxic effect on GBM cells [100]. In combination with oncolytic herpes simplex virus, the antitumor efficacy of the drug was enhanced in both immunodeficient and immunocompetent orthotopic GBM models [101]. In 2021, the combination of gemcitabine and axitinib was found to be cytotoxic for medulloblastoma cells. The same drug combination was well tolerated and tolerated in orthotopic human medulloblastoma xenograft mouse models [102]. One study in 2013 demonstrated the positive effects of axitinib in an organotypic in vivo model of human meningiomas [103] (Table 1).

### 3.2. Sorafenib

**Figure 4:**
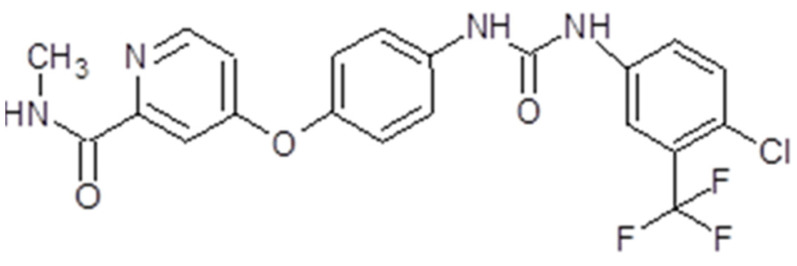
Sorafenib chemical structure. The design was created with ACD/ChemSketch 2.0 (Freeware).

Sorafenib, also known as Nexavar^®^ or BAY43-9006 (Bayer Pharmaceuticals Corp., West Haven, CT, USA and Onyx Pharmaceuticals Corp., Emeryville, CA, USA), is a TK inhibitor. It is a bi-aryl urea with a molecular weight of 464,825 g/mol (Figure 4). The drug was obtained through modification of the commercially available Raf-kinase inhibitor GK-00687. This small molecule is orally available and has multi-kinase inhibitory activity. This includes the capacity to inhibit pro-angiogenic RTKs like VEGFR2 [104]. Regarding its mechanism of action, the drug is capable of inhibiting RTKs like VEGFR, PDGFR or Raf in this way, impeding tumor growth and angiogenesis [105]. Also, it has been shown that sorafenib has the capacity to inhibit ERK signaling by reducing ERK phosphorylation. In nanomolar concentrations, it has an antiproliferative activity, while, in micromolar concentrations, it has antiproliferative activity [106]. The inhibition of proliferation was observed in human tumor cell lines that contained the mutations K-RAS or B-Raf [107] (Figure 3).

The presentation of sorafenib is tablets for oral administration. Taken orally, the drug exhibits a bioavailability of 38–49%. The maximum plasma concentration is achieved in about 3 h. Sorafenib is metabolized primarily in the liver through oxidative metabolism and glucuronidation. Its metabolites are found in plasma, feces and urine. The metabolism of the drug is not altered in patients with chronic liver disease or if the creatinine clearance is under 30 mL/min [108].

Preclinical studies have demonstrated the capacity of sorafenib to inhibit tumor growth in many solid tumors [106]. The first studied effect of the drug was on renal cancer patients. Also, sorafenib was shown to reduce proliferation, and induced apoptosis in GBM both in vitro and in vivo. One study in 2006 demonstrated that the drug had the capacity to inhibit the proliferation of GBM cell lines, but also acted synergistically with bortezomib [109]. In 2010, the efficacy of the drug was tested both in vitro and in vivo. It was observed that sorafenib determined a dose-dependent inhibition of proliferation of GBM cells, also inducing autophagy and apoptosis, and inhibited the phosphorylation of signal transducer and activator of transcription 3 (Stat3), but also the expression of cyclins D and E. It was able to reduce angiogenesis, and was well tolerated [110]. In the same year, Yang et al. found out that sorafenib inhibited STAT3 signaling, contributing to growth arrest and inducing apoptosis in GBM cells [111]. In another in vitro study, Carra et al. also showed that sorafenib had an inhibitory effect on GBM cell proliferation, induced apoptosis by downregulating Mcl-1, and had a selective induction of cell death [112]. In 2016, the investigators detected no radio-sensitization and no chemo-sensitization of the drug in GBM cell lines. The drug had no effect on double treatment with irradiation and temozolomide [113] (Table 1).

Regarding meningioma cells, one in vitro study in 2014 demonstrated that sorafenib targets meningioma cell motility and brain invasion [114] (Table 1).

### 3.3. Lenvatinib

**Figure 5:**
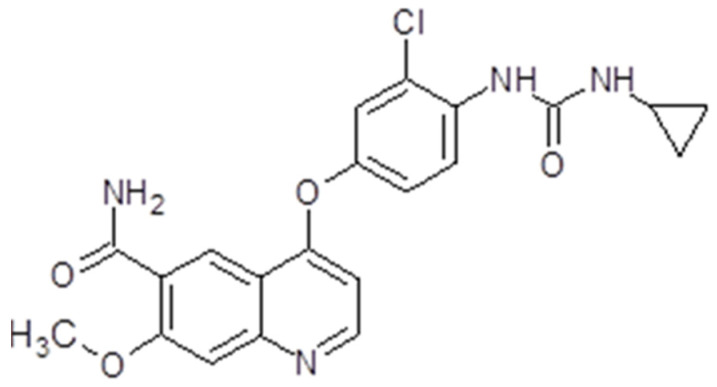
Lenvatinib chemical structure. The design was created with ACD/ChemSketch 2.0 (Freeware).

Lenvatinib, also known as Lenvima^®^, Kisplyx^®^ or E7080 (Eisai Co., Ltd., Tokyo, Japan), is a multiple TK inhibitor. The drug is a quinoline derivative with a molecular weight of 426,853 g/mol, and is used as a mesylate salt (Figure 5). It is orally available. Lenvatinib has the capacity to inhibit multiple tyrosine kinases like VEGFR1-3, FGFR1-4, PDGFR, RET or KIT [115]. The drug proved to be an angiogenesis inhibitor and have effects on tumor cell migration and invasion, but does not influence tumor cell proliferation [116] (Figure 3).

The presentation of lenvatinib is capsules for oral administration. It is absorbed rapidly and reaches maximum concentration in 1 to 4 h after ingestion. The bioavailability is 85–90%. After ingestion, the substance is bound to plasma proteins, albumin in particular, and is metabolized in the liver by the CYP3A4 enzyme. Mild-to-moderate liver impairment did not justify dose adjustment. In patients with severe liver damage, it is recommended to administrate 14 mg instead of 24 mg of lenvatinib [117]. The terminal half-life of the drug is 28 h. It is excreted via the feces (about two-thirds) and via urine (about a quarter) [118].

Several in vitro studies have demonstrated the anti-angiogenic effect of lenvatinib via inhibition of VEGFR2, VEGFR 3 and FGF1. Lenvatinib caused the regression of human small cell lung cancer cells through inhibition of angiogenesis [119]. It also inhibited VEGFR3-kinase in decreasing lymphatic vessel density within the metastatic lymph nodes after the resection of mammary breast tumor [115]. In one preclinical study in 2009, Ikuta et al. found that lenvatinib is capable of inhibiting the proliferation of endothelial cells and inhibited the progression of three cell lines of malignant pleural mesothelioma [120].

Lenvatinib has also been considered for brain tumor treatment. Regarding GBM, the first animal test was in 2017, when the drug was administrated to mice with advanced GB. In fact, the drug improved long-term survival [121] (Table 1).

### 3.4. Pazopanib

**Figure 6:**
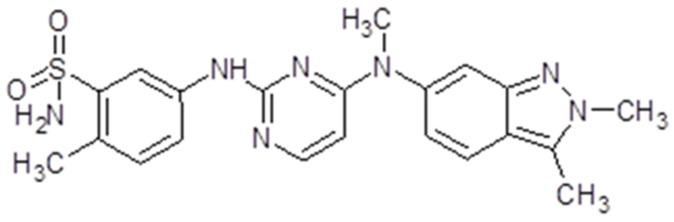
Pazopanib chemical structure. The design was created with ACD/ChemSketch 2.0 (Freeware).

Another multiple TK inhibitor is pazopanib (GW786034, Votrient^®^, Glaxo SmithKleine, Brentford, United Kingdom).The drug is part of the group of indazolyl pyrimidines. For commercial use is available a hydrochloride salt insoluble at ph ≥ 4 and lightly soluble at ph = 1 [122]. The molecular weight is 437,517 g/mol (Figure 6).

The presentation of pazopanib is tablet or oral suspension. The recommended dose is 800 mg. At this dosage, the maximum plasma concentration is reached after 2–4 h [123]. The drug is bound to plasma proteins like albumin or α1 glycoprotein up to 99.99% [124]. The bioavailability is 21.4%. Pazopanib is metabolized mainly by oxidation via CYP3A4 and additionally by glucuronidation. In patients with mild hepatic impairment, the drug clearance is 50% lower; therefore, in this situation, the dosage should be diminished. Pazopanib should not be administrated in patients with severe hepatic impairment [125]. There are seven metabolites of the drug and the excretion is primarily through the feces (82.2%) [126].

Pazopanib is capable of inhibiting VEGFR 1 and 2, PDGFR α and PDGFRβ, c-KIT and FGR-1, 3 and 4 [127]. Through its capacity to inhibit VEGF, pazopanib inhibits the FGF-induced proliferation of human umbilical vein endothelial cell (HUVEC) cultures in vitro, but also impairs VEGF-induced angiogenesis as well as FGF-induced angiogenesis in mouse corneal micropocket [127]. Also, the drug induced the inhibition of tumor growth in xenograft models and the inhibition of VEGFR-2 phosphorylation [127] (Figure 3).

### 3.5. Sunitinib

**Figure 7:**
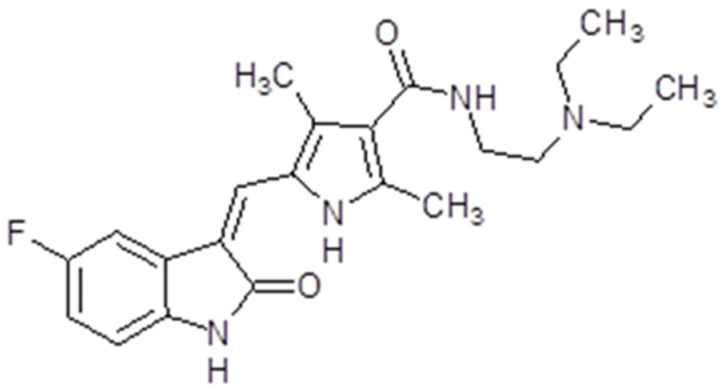
Sunitinib chemical structure. The design was created with ACD/ChemSketch 2.0 (Freeware).

Sunitinib (Sutent^®^ or SU11248, Pfizer Inc, New York, NY, USA) is also known as INN-sunitinib malate. Is a member of the pyrroles and is a monocarboxylic acid amide. The molecular weight is 398.474 g/mol (Figure 7). The presentation of sunitinib is oral capsules, while the recommended dose is 50 mg. The time to maximum plasma concentration is 6 to 12 h [128]. Sunitinib binds to human plasma protein. The bioavailability is not influenced by food intake. The drug is primarily metabolized by cytochrome P450 3A4 to an active *N*-desethyl metabolite, which is also metabolized by the cytochrome P450 3A4. The drug is mainly eliminated through feces and only 16% is found in urine [128].

Sunitinib is capable of inhibiting VEGFRs (VEGFR1, VEGFR2 and VEGFR3), PDGFRs (PDGFRα and PDGFRβ), Fms-like tyrosine kinase-3 receptor (FLT3), stem cell factor receptor (KIT) and the glial cell line-derived neurotrophic factor receptor (RET). This multi-target RTK inhibitor has an anti-angiogenic effect through VEGFR1, VEGFR2 and PDGFRβ-diminished signalization. It also has a potent antitumor activity on a variety of solid tumors. In preclinical studies, sunitinib demonstrated positive effects and tumor regression in various cancer cells, mainly gastrointestinal stromal tumors and advanced renal cell carcinoma [129] (Figure 3).

The anti-angiogenic effects of sunitinib were tested on orthotopic models of GBM in 2007. The drug had potent anti-angiogenic activity and prolonged survival [130]. In 2015, D’Amico et al. created a murine model of GBM expressing PDF-IRES-Cre retrovirus. The authors demonstrated that the combination of sunitinib and high-dose radiation was not only well tolerated but also delayed tumor growth. The association between sunitinib and low-dose radiation did not improve survival [131]. Another preclinical study was published in 2017. The authors demonstrated that the combination of CXCR4 antagonist and PRX177561 with sunitinib and bevacizumab inhibited tumor growth in preclinical models of human GBM [132]. In 2023, the results of a study that evaluated the activity of 16 new sunitinib derivatives in brain cancer cells and spheroids were published. The drug was demonstrated to have a significant impact on spheroid growth [133]. Ho et al. reported the results of a study on the combination of sunitinib and guanabenz in 2021. The effect of the angiogenenesis inhibitor on GBM cells was enhanced by guanabenz. Similar results were obtained on xenograft mice [134] (Table 1). For patients diagnosed with meningiomas, sunitinib had cytostatic and anti-migratory effects on human meningioma cells [135] (Table 1). In 2016, sunitinib was tested in a murine model with plexiform neurofibromatosis. The results were rather encouraging [136] (Table 1).

### 3.6. Cabozantinib

**Figure 8:**
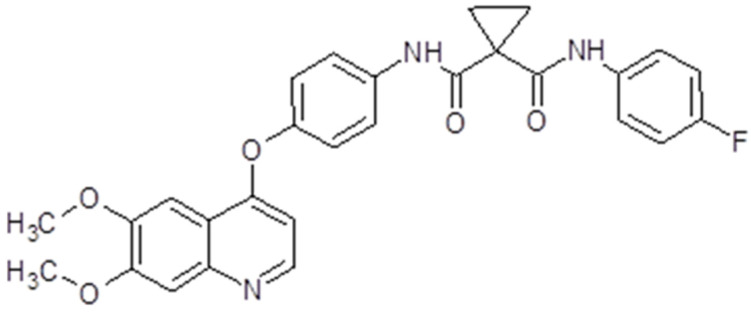
Cabozantinib chemical structure. The design was created with ACD/ChemSketch 2.0 (Freeware).

Cabozantinib (Cabometyx^®^, Cometriq^®^, Exelixis, Inc., Alameda, CA, USA) has a molecular mass of 501.514 g/mol (Figure 8). The presentation is oral capsules containing 20 mg, 40 mg or 60 mg of cabozantinib (S)-malate. The maximum plasma concentration is reached in 3 to 4 h, with a mean plasma concentration at a 60 mg dose [137]. Its clearance is rather variable. The drug is metabolized mainly by the P450 (CYP) 3A4 pathway but also by CYP2C9. The terminal half-life of the drug is 90–120 h. Therefore, hepatic impairment, medication or food intake may influence cabozantinib plasma concentrations [138].

The drug proved to be a potent inhibitor of VEGFR2 and MET, but also Flt3, RET, KIT and AXL. The activation of multiple RTKs initiates downstream signaling pathways like PI3K/AKT, MAPK or JAK/STAT [139]. Unlike other anti-angiogenic multi-target TKIs, cabozantinib has a greater potency, making it a promising agent for investigation (Figure 3). As a result, it has been tested both in vitro and in vivo in solid tumors like medullary thyroid cancer [140], prostate cancer [141], osteosarcoma [142], schwannoma [143], gastrointestinal stromal tumor [144], pancreatic neuroendocrine tumors [145] and glioblastoma [146]. These in vitro and in vivo results led to clinical trials, which finally resulted in cabozantinib’s FDA approval for several solid tumors.

Cabozantinib (XL184) suppressed angiogenesis, cellular invasion and tumor growth in GBM cells [139]. The drug effects were also tested on c-MET-positive orthotopic E98 glioblastoma xenografts and it is a promising therapy for c-MET-positive glioma [146] (Table 1).

In meningiomas, cabozantinib inhibits the VEGFR2 and MET signaling pathways. In 2021, a clinical case was reported of regression of intracranial meningiomas after cabozantinib administration [147]. A phase II study of cabozantinib for patients with recurrent or progressive meningioma is now recruiting (Table 1).

### 3.7. Nintedanib

**Figure 9:**
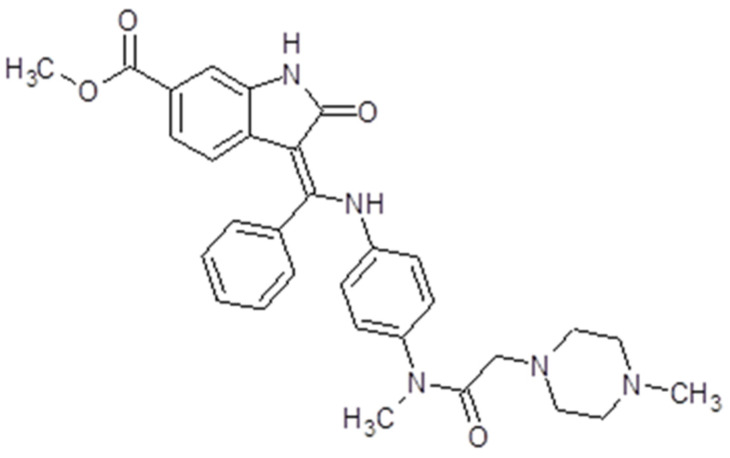
Nintedanib chemical structure. The design was created with ACD/ChemSketch 2.0 (Freeware).

Nintedanib (Ofev^®^, Boehringer Ingelheim, Biberach, Germany) or Vargatef, also called BIBF1120, is a salt with ethanesulfonic acid. The molecular weight is 539.6248 g/mol (Figure 9). The presentation is 100 mg capsules for oral use. The bioavailability is 4.7%. The peak plasma concentration is reached in 2 to 4 h, while the inactivation is due to esterases. The drug is mostly excreted via bile and feces. Because it is a substrate for P-glycoprotein, several drugs influence its action either by diminishing or increasing it [148].

As a multi-target tyrosine kinase inhibitor, nintedanib inhibits both PDGFR receptors along with VEGFR 1, 2, 3 and Flt3, but also FGFR 1, 2 and 3. Therefore, the drug initiates some downstream signaling pathways like PI3K/AKT, Ras/Raf/MEK/MAPK or FAK/Paxilin, inhibiting cell proliferation and angiogenesis [149] (Figure 3).

Nintedanib has been tested in vitro and in vivo on several solid tumors like lung adenocarcinoma [150], osteosarcoma [151], prostate adenocarcinoma [152], colorectal and hepatocellular carcinoma, as well as in gynecological tumors [153]. Although the drug has potential to be used in treatment of several tumors, its poor bioavailability is still a challenge. In 2025, Dang et al. investigated the effect of nintedanib on GBM cells and its mechanism of action. The inhibitor had a significant inhibitory effect on GBM cells and the drug delivery through the BBB was optimized through nanotechnology [154].

### 3.8. Regorafenib

**Figure 10:**
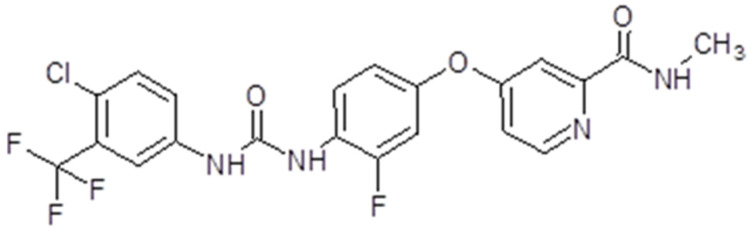
Regorafenib chemical structure. The design was created with ACD/ChemSketch 2.0 (Freeware).

Regorafenib (BAY-73 4506, Stivarga^®^, Bayer AG, Leverkusen, Germany) is a diphenylurea. The molecular weight is 482,82 g/mol (Figure 10). The presentation is 40 mg tablets for oral use with a bioavailability of 69–83%. The peak plasma concentration is reached in about 4 h after ingestion. Regorafenib is metabolized primarily in the liver. The result is two major and six minor metabolites. The two major metabolites M-2 and M-5 are pharmacologically active [155]. It is recommended that the drug is taken with a low-fat meal [156]. The unbound regorafenib or its metabolites are hydrolyzed in the enterohepatic circulation by the microbial actors in the gastrointestinal tract and then reabsorbed [157].

Regorafenib is a multi-target TKs inhibitor that targets receptor tyrosine kinases like VEGFR 1,2,3; TIE2; PDGFRβ; FGFR; KIT; REF and RAF. Therefore, the drug has anti-angiogenic, anti-metastatic and immunomodulatory effects [157] (Figure 3). It has been tested in vitro and in vivo on solid tumors like neuroblastoma [158], lung squamous cell carcinoma [159], osteosarcoma [160] and colorectal cancer [161].

Several preclinical studies have demonstrated the effect of regorafenib on glioma stem cells. In fact, the drug was able to determine a dose-dependent reduction of GSC’s pro-angiogenic ability [162,163]. Regorafenib’s dependent autophagy has also been reported [164], as well as apoptosis [165]. In some in vivo studies, regorafenib was able to inhibit tumor vascularization in GBM xenograft models. Also, the drug proved its anti-angiogenic and antitumor effects in GBM xenograft models [161,164]. A preclinical in vitro and in vivo study in 2017 established that regorafenib targets PDGFR and p44/42 ERK signaling. It also reduced cell motility and invasion, and mice with orthotopic meningioma xenografts presented a diminished tumor volume [166] (Table 1).

### 3.9. Aflibercept

Aflibercept (Eylea^®^, Zaltrap^®^, Regeneron Pharmaceuticals Inc., Westchester County, NY, USA) is a human monoclonal antibody. The molecular weight is 96.9 kilo Daltons (kDa). It is a dimeric glycoprotein. The VEGF-binding portion from the extracellular domains of human VEGF receptors 1 and 2 fused to the Fc portion of a human IgG1 immunoglobulin and formed a recombinant fusion protein [167], in this way acting as a VEGF-Trap. Aflibercept has a high affinity for VEGF A, B and PlGF (placental growth factor), and a strong bonding affinity to VEGFR. Also, it has the capacity to inhibit VEGF-A, VEGF-B, PIGF-1 and PIGF-2, as a consequence blocking their interaction with the cognate receptors and finally inhibiting angiogenesis [168] (Figure 3).

In cancer patients, the drug is administered 4 mg/kg intravenously every 2 weeks. It does not interfere with hepatic impairment, while renal dysfunction has no effect on Aflibercept clearance. The effects of the drug on various solid tumors have been tested in vitro and in vivo. Aflibercept’s effects have been investigated on tumors like retinoblastoma [169] and colorectal cancer [170].

One in vitro study demonstrated that VEGF-Trap treatment associated with radiation therapy significantly reduced tumor growth in a U87 subcutaneous xenograft model [171]. It was followed by an in vivo study in 2008 which reported that aflibercept therapy significantly prolonged the survival of glioma xenograft-bearing mice [172] (Table 1).

### 3.10. Ramucirumab

Ramucirumab (Cyramza^®^, Elli Lilly&Co, Indianapollis, IN, USA), also known as IMC-1121B, or LY3009806, is a human monoclonal antibody. The molecular weight is 143.6 kDa. The drug is a direct antagonist of VEGFR2. Therefore, by binding a specific epitope on the extracellular domain of VEGFR2, the drug blocks the binding of the receptor ligands (VEGF-A, VEGF-C and VEGF-D). In this way, the VEGF-stimulated phosphorylation of the receptor is prevented. Events like downstream ligand-induced proliferation, migration and permeability of human endothelial cells are no longer present. VEGF-mediated angiogenesis is inhibited [173] (Figure 3). The drug is administered intravenously 8 mg/kg up to 10 mg/kg depending on the regimen used. The clearance decreases as the dose of ramucirumab increases. The mean half-life at 8 mg/kg dose is 123 h for the first infusion and 318 h for the last infusion [174]. Renal deficiency or hepatic damage do not influence the drug dosage. Ramucirumab has in vitro and in vivo inhibitory effects on leukemia and ovarian cancer cell lines [154]. Also, in combination with paclitaxel, the monoclonal antibody was able to enhance the inhibitory effect of paclitaxel in gastric cancer cell lines [175]. No preclinical studies have been performed until now on brain tumors cells.

**Table 1:** Main in vitro and in vivo studies that demonstrate the efficacy of anti-angiogenic multi-target therapies on brain tumors.

Study	Study Area	Materials	Signaling Pathway	Molecular Mechanism
Lu et al., 2015 [97]	United States of America	Human glioma cells, Human GSCs, Human umbilical vein endothelial cells, Human brain microvascular endothelial cells, mice	No signaling pathway mentioned VEGFRs/PDGFR	Axitinib exhibits anti-angiogenic activity and prolongs survival of mice bearing orthotopic GBMs.
Morelli et al., 2016 [98]	Italy	Human glioma cells	No signaling pathway mentioned	Axitinib induces DNA damage response (DDR) characterized by γ-H2AX phosphorylation and Chk1 kinase activation leading to G2/M cell cycle arrest and mitotic catastrophe in glioma cell lines. Combined exposure to axitinib and bortezomib was more effective in inhibiting cell viability of all glioma cell lines.
Krcek et al., 2017 [99]	Germany	Human glioma cells	MAPKAP VEGFR2/phospholipase C/protein kinase C/ERK	The combination of axitinib and irradiation could be a potent strategy in the treatment of GBM.
Oprita et al., 2023 [100]	Romania	Human glioma cell line	No signaling pathway mentioned	Axitinib and sorafenib retarded GB1B cell growth in terms of dose and duration.
Saha et al., 2018 [101]	United States of America	GFP-positive mouse, Mouse brain microvascular endothelial cells (MBMECs), Human primary and recurrent GSCs	PDGFR/ERK/Akt	Axitinib has a dose-dependent anti-angiogenic effect while the antitumor effects of axtinib + G47Δ-mIL12 were mainly T-cell dependent.
Schwinn et al., 2021 [102]	Germany	Human medulloblastoma cells, mice	No signaling pathway mentioned	The combination of axitinib and gemcitabine has cytotoxic effects on medulloblastoma cells and favorable tolerability in xenograft models.
Yu et al., 2006 [109]	United America	Human glioma cells	Akt	Sorafenib interacts synergistically with bortezomib to induce apoptosis in glioma cells.
Siegelin et al., 2010 [110]	United States of America	Established or human glioma cells, mice	PI3K/AKT	Sorafenib has potent anti-glioma activity in vitro and in vivo.
Yang et al., 2010 [111]	United States of America	Human GBM cells	Akt, MAPK	The inhibition of STAT3 signaling by sorafenib contributes to growth arrest and induction of apoptosis in glioblastoma cells.
Carra et al., 2013 [112]	Italy	Human GBM cells	MAPK PI-3/Akt	Sorafenib reduces proliferation of glioblastoma cultures, and this effect depends, at least in part, on the inhibition of PI3K/Akt and MAPK pathways. Sorafenib significantly induces apoptosis/cell death via downregulation of the survival factor Mcl-1. Sorafenib has a selective action on glioblastoma stem cells.
Riedel et al., 2016 [113]	Germany	Human GBM cells	MAPK, Akt	Sorafenib had only minor effects on cell survival when administered alone and failed to enhance GBM cell killing by irradiation, TMZ or combined treatment, and instead rather caused resistance in some cell lines.
Wilisch-Neumann et al., 2014 [114]	Germany	Human meningioma cells	MAPK PI-3/Akt	Sorafenib reduce meningioma cell motility and brain invasion.
Jia Li et al., 2017 [121]	China	Human glioma cells, nude mice	No signaling pathway mentioned	Lenvatinib significantly increased apoptosis in glioma cell lines, and tumor growth was significantly inhibited in tumor-bearing mice.
Bouard et al., 2007 [130]	France	GBM cell lines, intra-cerebral xenograft models	No signaling pathway mentioned	Sunitinib had potent anti-angiogenic activity and prolonged survival.
D’Amico et al., 2012 [131]	USA	mice	No signaling pathway mentioned	The addition of sunitinib to radiotherapy enhances the effects of radiation in the brain and delays GBM growth without altering overall survival at the studied doses.
Gravina et al., 2017 [132]	Italy	GBM cells, subcutaneous xenografts, intracranial xenografts	No signaling pathway mentioned	An enhanced survival effect on GBM-bearing mice which were treated with a combination of PRX177561 and bevacizumab or sunitinib
Andrae et al., 2012 [135]	Germany	Human meningioma cells	PI3K/AKT ERK	Sunitinib strongly reduced meningioma cell migration in vitro, and had cytostatic effects.
Dang et al., 2025 [154]	China	GBM cells	No signaling pathway mentioned	Nintedanib exerted significant inhibitory effects on GBM cells. Drug delivery through nanotechnology may represent a new strategy for GBM treatment

## 4. Clinical Trials Assessing the Effectiveness of FDA-Approved Anti-Angiogenic Inhibitors

### 4.1. Axitinib

The first clinical studies using axitinib were conducted on patients diagnosed with refractory solid tumors. The result of this phase I study determined the dose of 5 mg twice a day as the recommended dose for the next trials. The limited dosage was the result of toxicities like arterial hypertension [176]. It was followed by phase II and III clinical trials that tested the drug on patients diagnosed with kidney cancer [83,177,178]. As a result, in 2012, FDA approved axitinib for patients with advanced or metastatic clear cell carcinoma who had failed on one previous regimen therapy [86].

Following the promising preclinical results, a number of clinical trials studied the effect of axitinib on patients diagnosed with GBM. In 2019, Duernick et al. completed and published the results of a randomized phase II clinical trial which compared the effect of the drug as a single agent with the combination of axitinib and lomustin in patients diagnosed with recurrent GBM. Monotherapy with axitinib proved to be more efficient in improving the response rate and the PFS in recurrent GBM patients [179]. In 2020, Awada et al. published the results of another single-center phase II clinical trial (GliAvAx) with axitinib and avelumab for patients with recurrent GBM. Patients with prior treatment (surgery, radiation therapy, chemotherapy with temozolomide) were split into two groups in accordance with the dose of corticoids received. Those who had a daily dose of corticoid under 8 mg received the drug combination. Those who had a daily dose of corticoid of more than 8 mg, initiated therapy with axitinib and avelumab was added only if the corticoid therapy could be tapered under 8 mg. Although the combination of the two drugs had an acceptable toxicity, the clinical trial failed to meet its primary objective [180] (Table 2).

A phase II study of axitinib in patients with neurofibromatosis type 2 and progressive vestibular schwannomas showed that the drug had rather modest antitumor activity and greater toxicity when compared with bevacizumab [181].

**Table 2:** Main clinical studies that used anti-angiogenic multi-targeted therapy for glioma treatment.

Trial Reference	Year	Who Tumor Grade, Histology Reference	Number of Patients	Clinical Trial Phase	Regimen	Signaling Pathways	Endpoints	Systemic Toxicity and Other Adverse Events
Duernik et al. [179]	2018	IV, rGBM	79	II	Axitinib 5 mg twice daily. Axitinib 5 mg twice daily and Lomustine 130 mg/m^2^ orally as a single dose every 6 weeks	not mentioned	6 m PFS OS	neutropenia in the axitinib plus lomustine arm
Awada et al. [180]	2020	IV, rGBM	54	II	Axitinib 5 mg twice daily and avelumab 10 mg/kg intravenous every 2 weeks for patients with a daily dose of ≤8 mg of methylprednisolone. Patients with a higher dose of corticotherapy started with Axitinib 5 mg twice daily and added Avelumab 10 mg/kg intravenous every 2 weeks after 6 weeks if the corticotherapy dosage reached ≤8 mg.	not mentioned	6 mPFS OS	dysphonia lymphopenia arterial hypertension diarrhea
Reardon et al. [182]	2011	IV, rGBM	32	II	Sorafenib 400 mg twice daily and temozolomide 50 mg/m	not mentioned	PFS-6 OS Toxicity of sorafenib and temozolomide The pharmacokinetics of sorafenib when combined with daily temozolomide	grade 2 and 3 elevation of amylase or lipase occurred in 2 and 4 patients grade 2 and 3 palmar-plantar erythrodysesthesia (PPE) occurred in 1(3%) and 6 (19%) patients fatigue rash infection electrolyte disturbances intracranial hemorrhage
Peereboom et al. [183]	2013	IV rGBM	24	II	Sorafenib 200 mg twice daily and tipifarnib 200 mg twice daily	Ras/Raf/Mek/ERK	Define DLT and determine the MTD	fatigue lipase diarrhea nausea pain rash AST ALT
Den et al. [184]	2014	WHO III grade or GBM	15	I	Radiation therapy consisted of a conventionally fractionated regimen to a total dose of 60 Gy, administered in 30 daily fractions of 2 Gy, with or without volumetric modulated arc therapy. Cohort 1 received a single daily oral dose of 200 mg, cohort 2 received 200 mg Sb BID and cohort 3 received 400 mg. After a break of 4 weeks, patients were treated in the maintenance phase with TMZ (150 mg m^−2^ on d1–5 for the first cycle of 28 days) followed for a total of up to six cycles of TMZ given at 200 mg m^−2^ on d1–5/28 if the first cycle was tolerated without significant side effects. Sorafenib restarted with 400 mg daily.	MAPK	The safety profile and tolerability of Sb when administered in conjunction with TMZ and RT and to establish the MTD of this combination. Secondary objectives were to evaluate pharmacokinetics (PKs), tumor response and survival.	thrombocytopenia fatigue hand–foot skin reaction
Nabors et al. [185]	2011	GBM, anaplastic astrocytoma, or anaplastic oligodendroglioma	47	I	Sorafenib 400 mg twice daily	Ras/Raf/MAPK	MTD	dermatological toxicity fatigue hyperglycemia hypertension hypophosphatemia nausea back pain joint pain.
Hottinger et al. [186]	2014	WHO grade III or GBM	15	I	Radiation therapy consisted of a conventionally fractionated regimen to a total dose of 60 Gy, administered in 30 daily fractions of 2 Gy, with or without volumetric modulated arc therapy. Three dose levels for Sb were planned as follows: cohort 1 received a single daily oral dose of 200 mg, cohort 2 received200 mg Sb and cohort 3 received 400 mg. After a break of 4 weeks, patients were treated in the maintenance phase with TMZ (150 mg m 2 on d1–5 for the first cycle of 28 days) followed for a total of up to six cycles of TMZ given at 200 mg m 2 on d1–5/28 if the first cycle was tolerated without significant side effects. Sorafenib was restarted on day 1 of the first cycle at 400 mg.	MAPK	The safety and maximum tolerated dose (MTD) of Sb in combination with radiation therapy (RT) and temozolomide (TMZ)	thrombocytopenia fatigue hand–foot skin reaction skin rush dyslipidemia diarrhea hypertension heart rate abnormalities
Chen et al. [187]	2020	GBM or gliosarcoma	57	I/II	Patients initially received sorafenib at 200 mg BID and erlotinib at 100 mg QD.	Ras/Raf/MAPK	MTD of sorafenib + erlotinib, charac terization of toxicities, and evaluation of drug interactions via pharmacokinetics studies. 6-month PFS (PFS6)	lymphocyte count decreased hypophosphatemia fatigue diarrhea lipase increased abdominal pain arthralgia dysphasia
Reardon et al. [188]	2012	rGBM	32	II	24 mg Levantinib once daily in 28 cycles	Not mentioned	PFS-6	hypertension fatigue headache proteinuria diarrhea fatigue hypertension one patient died due to pulmonary embolism
Lwin et al. [189]	2020	GBM	31	II	len 20 mg/d + pembro 200 mg Q3W for 35 cycles or until confirmed PD	Not mentioned	Efficacy and safety of lenvatinib plus pembro in pts with previously treated advanced solid tumors. Secondary endpoints included disease control rate (DCR), duration of response (DOR), PFS, and OS	manageable toxicity
Iwamoto et al. [190]	2010	rGBM	35	II	Pazopanib 800 mg orally daily on 28-day cycles	Not mentioned	PFS6 OS	anemia leukopenia lymphopenia neutropenia thrombocytopenia arterial hypertension fatigue excessive sweating weight loss decubitus ulcer dry skin flushing hand–foot syndrome hypopigmentation pruritus anorexia constipation diarrhea abdominal distension flatulence gastritis heartburn nausea CNS hemorrhage epistaxis elevated ALT elevated AST hyperbilirubinemia hypermagnesemia hypoalbuminemia hypophosphatemia joint or limb pain proteinuria abdominal pain thromboembolic event
Reardon et al. [191]	2013	rGBM	75	I/II	Pazopanib 400 mg q.d. plus lapatinib 1000 mg	Not mentioned	6-month PFS Pharmacokinetics Maximum observed C Concentration time to maximum concentration Concentration 24 h post-dose	diarrhea fatigue hypertension nausea elevated ALT thrombocytopenia neutropenia elevated AST rash
Burzynski et al. [192]	2014	GBM	11	preliminary	Pazopanib 200 mg/daily–400 mg/daily Everolimus 5–10 mg/daily Sirolimus 1–3 mg daily Dasatinib 50 mg/daily Vorinostat 200–300 mg/daily Erlotinib 100–150 mg/daily Lapatinib 750 mg/daily Bevacizumab 2.5–10 mg/daily	Not mentioned	Further phase I/II clinical trials with PB in combination with pazopanib, dasatinib, everolimus and BVZ in patients with RGBM who failed standard surgery, radiation therapy and chemotherapy	anemia leukopenia thrombocytopenia hypertension fatigue sweating (diaphoresis) rash diarrhea dysphagia mucositis/stomatitis (clinical exam) hemorrhage, CNS alkaline phosphatase hyponatremia proteinuria neuropathy: sensory (paresthesia) pain: neck
Saada et al. [193]	2024	GBM	35	I/II	800 mg orally daily on 28-day cycles	Not mentioned	PFS6 OS	hypertension increase ALT asthenia nausea diarrhea thrombopenia neutropenia anemia
Wuthrick et al. 2011 [194]	2011	Primary brain tumors and metastatic central nervous system malignancies	15	I	37.5 mg sunitinib	Not mentioned	PFS OS	leukopenia thrombocytopenia anemia lymphopenia neutropenia hyponatremia hyperglycemia hypocalcemia hypercarbia hyperuremia hypokalemia elevated creatinine hyperbilirubinemia hypoproteinemia hypoalbuminemia elevated Alk. Phosphatase elevated ALT elevated AST fatigue pain dermatitis chest pain alopecia edema nausea anorexia diarrhea dyspepsia emesis mucositis seizure dysphasia drooling/difficulty chewing headache motor neuropathy DVT pulmonary embolism dyspnea epistaxis vaginal bleeding hypertension
Neyns, et al. [195]	2011	HGG	21	II	37.5 mg sunitinib	Not mentioned	PFS OS	skin toxicity neutropenia thrombocytopenia lymphocytopenia
Duernick et al. [196]	2015	Anaplastic or low-grade glioma	13	II	Sunitinib malate (Sutent, Pfizer) was administered at a daily dose of 25 mg for 28 consecutive days followed by a 14-day treatment-free interval. Lomustine (CCNU) was administered as a single dose (80 mg/m^2^) on day 14 of the 6-week treatment cycle	Not mentioned	PFS OS	fatigue mucositis thrombocytopenia lymphopenia neutropenia
Wetmore et al. [197]	2016	Recurrent or refractory high-grade glioma or ependymoma	30	II	Sunitinib, 15 mg/m^2^	Not mentioned	PFS OS	alanine aminotransferase increased aspartate aminotransferase increased lipase increased lymphocyte count decreased neutrophil count decreased serum amylase increased white blood cell decreased diarrhea fatigue intracranial hemorrhage rash maculo-papular skin and subcutaneous tissue disorders—Other (rash, acne)
Wuthrick et al. [198]	2014	Recurrent high-grade glioma	11	I	37.5 mg sunitinib The fSRT doses delivered ranged from 30 to 42 Gy in 2.5- to 3.75-Gy fractions	(PI3K)-Akt-mTOR	Safety and toxicity profile of continuous daily-dosed sunitinib when combined with hypofractionated stereotactic RT (fSRT) for recurrent high-grade gliomas (rHGG).	leukopenia anemia thrombocytopenia fatigue candidiasis nausea vomiting diarrhea hypocalcemia hyponatremia acid reflux xerostomia hypoproteinemia hypochloremia elevated ALT elevated AST pain cough alopecia aphasia stomatitis anorexia hyperglycemia elevated ALP muscle weakness esophagitis hypertension
Faye et al. [199]	2023	*MGMT* promoter GBM	37	II	12.5 mg of daily sunitinib for 7 days, followed by concurrent chemoradiation plus 12.5 mg sunitinib, then adjuvant TMZ	Not mentioned	PFS OS safety	fatigue anemia leukopenia lymphocytopenia neutropenia thrombocytopenia pulmonary embolism deep vein thrombosis appetite loss (anorexia) constipation diarrhea dysgeusia (taste alteration) increased liver enzyme increased creatinine hyperglycemia nausea vomiting (emesis) weight loss (anorexia) seizures speech impairment ataxia muscle atrophy/weakness neuropathy cognitive disturbance confusion mood (depression/anxiety) dizziness drowsiness headache fever brain infection alopecia hypertension tachycardia coughing dyspnea shortness of breath on exertion
Janssen et al. [200]	2024	Recurrent GBM	55	II/III	High-dose intermittent sunitinib 300 mg once weekly (Q1W, part 1) or 700 mg once every two weeks (Q2W, part 2) or lomustine	Not mentioned	PFS OS	blurred vision diarrhea dysgeusia fatigue flu-like symptoms headache hypertension hypothyroidism mucositis oral muscle weakness lower limb musculoskeletal disorders nausea oral pain palmar–plantar erythrodysesthesia syndrome rash septic bursitis skin discoloration syncope tooth infection vertigo vomiting anemia lymphocyte count decreased neutrophil count decreased platelet count decreased white blood cell decreased alanine aminotransferase increased alkaline phosphatase increased aspartate aminotransferase increased GGT increased
Kaley et al. [201]	2014	Anaplastic meningioma	36	II	Sunitinib was administered at 50 mg/d for days 1–28 of every 42-day cycle	Not mentioned	PF6 Radiographic response safety	CNS hemorrhage thrombotic microangiopathy neutropenia hypophosphatemia fatigue thrombocytopenia lymphopenia leukopenia hypertension headache ALT AST dehydration pain, abdomen hyperglycemia rash, hand-foot reaction vomiting pancreatitis hypocalcemia confusion diarrhea creatinine hypomagnesemia prolonged QTc interval right ventricular enlargement thrombosis/embolism hyperuricemia gastrointestinal perforation
Cardona et al. [202]	2019	WHO II or WHO III meningiomas	31	II	Octreotide acetate LAR [O]/everolimus [E] (30 mg IM q28 days/10 mg PO q/day), sunitinib [Su] (50 mg PO q/day for days 1–28 of 42 days) or bevacizumab [Bev] (10 mg/kg IV days 1 and 15)	Not mentioned	PFS OS	fatigue hypothyroidism
Schiff et al. [203]	2016	HGG	26	I	Cabozantinib at a dose of 40 mg or 60 mg daily	MET	Grade 3/4 adverse events Maximum tolerated dose	thrombocytopenia fatigue constipation nausea diarrhea elevated ALT neutropenia elevated AST leukopenia elevated LDH hypertension
Cloughesy et al. [204]	2018	Progressive GBM	152	II	Cabozantinib starting dose of 140 mg/day, but the starting dose was amended to 100 mg/day because of toxicity.	MET	PF6 OS	fatigue diarrhea decreased appetite PPES nausea headache constipation hypertension weight decreased dysphonia AST increased ALT increased convulsion LDH increased hypophosphatemia confusional state stomatitis vomiting abdominal pain thrombocytopenia pain in extremity insomnia gait disturbance hair color changes leukopenia lipase increased cough dysgeusia anxiety oral pain depression dry skin hemiparesis dyspepsia edema peripheral oropharyngeal pain rash hypokalemia neutropenia dyspnea dizziness cognitive disorder lymphopenia proteinuria aphasia vision blurred bilirubin increased epistaxis skin discoloration
Muhic et al. [205]	2013	Recurrent GBM	13	II	Nintedanib was given orally at a dose of 200 mg twice daily	Not mentioned	PF OS	fatigue loss of appetite, diarrhea nausea
Norden et al. [206]	2015	HGG	36	II	Nintedanib was given orally at a dose of 200 mg twice daily	Not mentioned	PF OS	treatment was well tolerated
Lombardi et al. [207]	2019	Relapsed GB	124	II	Regorafenib 160 mg once daily for the first 3 weeks of each 4-week cycle or lomustine 110 mg/m^2^ once every 6 weeks	Not mentioned	PFS OS toxicity	hand–foot skin reaction increased lipase blood bilirubin increased
Chiesa et al. [208]	2022	Recurrent GBM	30	II	Regorafenib was administered orally at a dose of 160 mg/day for the first 3 weeks of each 4-week cycle	MAPK pathway	PFS OS	thrombocytopenia fatigue hand–foot syndrome diarrhea hyperbilirubinemia
Rudà et al. [209]	2022	Recurrent GBM	66	I/II	Regorafenib daily dose was gradually increased from 80 to 160 mg across the first 2 cycles.	Not mentioned	PFS OS toxicity	grade 3–4 toxicity
Fasano et al. [210]	2023	Recurrent GBM	56	II	160 mg of regorafenib (four 40 mg tablets) per day orally for three weeks in a four-week cycle	Not mentioned	PFS OS	hand–foot skin reaction rash/desquamation piastrinopenia neutropenia hypertension fatigue voice changes vomiting hepatic AEs, aspartate aminotransferase elevation hyperbilirubinemia proteinuria fever cardiac diarrhea
Nayak et al. [211]	2011	HGG	59	I	Aflibercept 4 mg/kg every 2 weeks	VEGF PIGF	Maximum tolerated dose Toxicities	abdominal pain Alanine aminotransferase increase Aspartate aminotransferase increase alkaline phosphatase increase bilirubin increase Gamma glutamyl transferase increase arthralgia colonic perforation colitis dehydration fatigue headache hypertension hypokalemia hyponatremia seizure nausea lung infection peripheral nerve infection 1 urinary tract infection vascular access complication leukopenia lymphopenia neutropenia thrombocytopenia
De Groot et al. [212]	2011	Malignant glioma	58	II	Aflibercept 4 mg/kg was administered intravenously on day 1 of every 2-week cycle	VEGF PIGF	PF6 Overall radiographic response	ataxia CNS ischemia confusion dysphagia fatigue GI hemorrhage hand–foot syndrome headache hypertension hyponatremia hypophosphatemia hypoxia increased LFTs lymphopenia mucositis neutropenia pain pericarditis proteinuria rash thrombosis/embolism wound complication

### 4.2. Sorafenib

Sorafenib treatment showed promising results in the phase I [213], phase II [214] and III [215] clinical trials, which led in 2005 to FDA approval of sorafenib for advanced renal cancer treatment. After that, in 2007, sorafenib was approved by the FDA as first-line treatment for patients with advanced HCC [216], while in 2013 the FDA approved sorafenib for the treatment of radioactive iodine-resistant (RAI-R) metastatic well-differentiated thyroid cancer (DTC) [217].

The drug was tested in clinical trials for some other solid tumors like desmoid tumors [218], pancreatic cancer [219], on behalf of the Italian Group for the Study of Digestive Tract Cancer (GISCAD), melanoma [220], non-small cell lung cancer (NSCLC) [221] and breast cancer [222].

A phase I clinical trial in 2011 determined the maximum tolerated dose of sorafenib in patients diagnosed with brain tumors (GBM, anaplastic astrocytoma or anaplastic oligodendroglioma), progressive or recurrent, after radiation therapy with or without chemotherapy. The maximum tolerated dose (MTD) of sorafenib given orally on a continuous basis was established as 600 mg in patients with malignant glioma who were concurrently receiving enzyme-inducing antiseizure drugs and 800 mg in those who were not [185]. However, when tested in clinical trials either alone or in combination with temozolomid or erlotinib, in patients with recurrent or progressive GBM, the effect of sorafenib was rather disappointing [182,183]. Regarding the association between sorafenib and X-irradiation there are some promising results [186]. A phase I/II clinical trial determined the MTD and 6-month PFS of patients diagnosed with GBM or gliosarcoma and treated with sorafenib and erlotinib. The results were rather disappointing [187] (Table 2). In 2014, a phase II study of sorafenib in children with recurrent or progressive low-grade astrocytomas also had disappointing results [223].

### 4.3. Lenvatinib

In clinical studies, lenvatinib demonstrated its efficiency for the treatment of several solid tumors. In one phase II clinical trial in 2015, Lenvatinib had a positive effect in patients with radiation-refractory differentiated thyroid cancer. The patients had a PFS of 12.6 months [224]. Also in 2015, a phase III clinical trial with lenvatinib was conducted in patients with radiation-refractory thyroid cancer with radiographic progression within the prior 12 months. In this situation, the median PFS was 18.3 months, and the toxicity was significant [225]. As a result, in 2015, the FDA approved lenvatinib for the treatment of radiation-refractory thyroid cancer [226]. The same drug in combination with everolimus was approved by the FDA in 2016 for the treatment of clear-cell renal carcinoma [227]. In 2018, lenvatinib received approval as a first-line treatment for patients with unresectable HCC [228]. In September 2019, the FDA approved the combination of pembrolizumab and lenvatinib for patients with certain types of endometrial carcinoma [229]. For several other solid tumors like melanoma [230], lung adenocarcinoma [231], ovarian cancer [232], breast cancer [233], biliary tract cancer [234] and pancreatic cancer [235] the results were also promising.

The first clinical trial that studied the effect of lenvatinib treatment on patients with progressive GBM on bevacizumab was in 2012. The results were rather modest: the PFS rate was 1.9 months, while the median overall survival (OS) was 4.11 months. Also, the tumor volume significantly decreased one day after the drug administration, which was considered to be a consequence of of lenvatinib’s capacity to decrease vascular permeability and tumor vascularity [188]. In 2020, the results of a phase II study of lenvatinib plus pembrolizumab were published. The association was demonstrated to have promising antitumor activity, and reduced toxicity. The median duration of response was 3.2 [189] (Table 2).

### 4.4. Pazopanib

The first FDA approval for pazopanib was in 2009 for the treatment of advanced renal cancer. This was a result of phase III clinical trials [236]. After that, the drug was approved by the FDA in 2012 for the treatment of soft tissue sarcoma [237].

Regarding GBM, a phase II trial of pazopanib did not prolong PFS in patients with recurrent GBM [190]. In 2013, Reardon et al. published the results of a phase I/II trial of pazopanib in association with lapatinib for patients with relapsed GBM. However, the results were rather disappointing, and the antitumor activity of this combination of drugs was limited [191]. In 2014, Burzynski et al. published another study of pazopanib in combination with phenylbutyrate in patients with GBM. The combination was tolerated. The conclusion was that further studies were needed [192].

The results of a phase I/II study of pazopanib and temozolomide in patients newly diagnosed with GBM were reported in 2024. This was the PAZOGLIO trial, of which the objective was to evaluate the safety of this drug combination. In fact, the patients were treated after the partial or complete resection of the tumor. The administration of the drug combination was carried out during the maintenance phase, as the Stupp regimen defines it. Saada et al. concluded that, after the evaluation of safety, which represents the first phase of the study, the association between temozolomide and pazopanib in a dose of 600 mg daily is feasible. However, phase II of the study, regarding the efficacy of the combination, is now enrolling patients [193] (Table 2).

### 4.5. Sunitinib

The first FDA approval for sunitinib was in 2006 for gastrointestinal stromal tumors and advanced renal cell carcinoma [238,239]. It was followed by the FDA approval of the drug for rare types of pancreatic cancer, which was a result of a phase III clinical trial [240,241].

A phase I clinical trial studied the effect of the combination of sunitinib and radiotherapy in patients with primary brain tumors. The drug had acceptable toxicity and the adverse events were limited. This study encouraged the researchers to progress to phase II clinical trials [194]. In 2011, Neyns et al. published the results of a phase II study of sunitinib malate in patients with recurrent high-grade glioma. The drug had insufficient activity [195]. Since then, there have been many clinical trials that have reported similar results. The drug was tested either as a single agent [242,243,244,245] or in combination with irinotecan [246]. Regarding the combination between sunitinib and lomustine, in 2015, Duernick et al. published the results of a clinical trial including 13 patients treated with this combination of drugs. Although this regimen was tolerated, it was not active enough [196]. Another phase II clinical trial in 2016 evaluated treatment with sunitinib for children diagnosed with recurrent or refractory high-grade glioma or ependymoma. The drug was sufficiently tolerated but did not have enough efficacy [197]. In 2014, a pilot study followed the effect of the combination of radiation therapy and sunitinib in previously irradiated recurrent high-grade glioma patients. The results were rather encouraging, with acceptable 6-month PFS [198]. In 2023, Faye et al. concluded after a phase II clinical trial that the concurrent administration of sunitinib, temozolomide and radiotherapy in newly diagnosed patients with MGMT unmethylated GBM might be beneficial [199]. In 2024, the STELLAR phase II/III clinical trial analyzed the intermittent administration of high-dose sunitinib in patients diagnosed with recurrent GBM. However, the conclusion was that this regimen failed to improve the outcome for the patients [200] (Table 2).

A phase II clinical trial of sunitinib for patients diagnosed with recurrent and progressive atypical and anaplastic meningioma demonstrated in 2014 that the drug is active in meningioma patients [201]. In 2019, a comparative survival and molecular marker analysis evaluated the efficacy of sunitinib and octreotide/everolimus on patients with malignant meningiomas. The results were similar [202] (Table 2).

### 4.6. Cabozantinib

The first FDA approval of cabozantinib was in 2017 for previously untreated advanced renal cancer carcinoma [247]. It was followed in 2019 by FDA approval of the drug for previously treated hepatocellular carcinoma [248]. In 2021, the association between nivolumab and cabozantinib was FDA approved as a first-line treatment for patients with advanced renal cancer carcinoma [249]. Also in 2021, the drug was approved for previously treated radioactive iodine-differentiated thyroid cancer [250].

A phase I trial of cabozantinib plus temozolomide and radiotherapy in patients newly diagnosed with high-grade glioma and pre-treated with radiation therapy established the dose of 40 mg daily. The drug was tolerated [203]. In 2018, a phase II clinical study of cabozantinib in patients with progressive GBM demonstrated a modest clinical activity [204] (Table 2).

A phase II study of cabozantinib for patients with recurrent or progressive meningioma is now recruiting (Table 1).

### 4.7. Nintedanib

The results of preclinical studies led to clinical trials for several solid tumors. Until now, the drug has not been FDA approved for any solid tumor. However, nintedanib received its first FDA approval in 2014 for the treatment of idiopathic pulmonary fibrosis [251]. In 2019, the drug received FDA approval for the treatment of interstitial lung disease associated with systemic sclerosis or scleroderma [252]. This was followed in 2020 by the FDA approval of nintedanib for treatment of chronic fibrosing interstitial lung disease with progressive phenotype [253].

Regarding GBM, one phase II open-label study determined that the single-agent nintedanib, at a dose of 200 mg twice a day, had a limited and clinically non-relevant antitumor activity [205]. Another phase II clinical trial in 2015 found that treatment with nintedanib, although tolerated, was not active against high-grade glioma, either prior to or after bevacizumab therapy [206] (Table 2).

### 4.8. Regorafenib

The result of preclinical studies was the investigation of regorafenib’s effect in a series of clinical trials, which resulted in the FDA’s approval of the drug for treatment of various tumors. The first FDA approval for regorafenib was in 2012 for patients with metastatic colorectal cancer [254]. The same year, the drug was approved for patients with advanced colorectal cancer. Next, the drug received approval to be used for advanced gastrointestinal stromal tumors in 2013 [238]. Since 2017, regorafenib has received approval to be used for hepatocellular carcinoma patients [255].

REGOMA is a clinical trial of which the aim was to compare regorafenib with lomustin for GBM patients. The drug significantly improved OS [207]. Similar results were obtained in 2022 by Chiesa et al. [208]. In another study, Ruda et al. reported the efficacy of the drug with improvement in PFS and OS [209]. Regorafenib had similar efficiency in elderly GBM patients [210]. A couple of studies followed the MRI evolution of GBM in patients treated with regorafenib. The conclusion was to assess the therapy with caution [256]. The drug was also evaluated in a phase I clinical trial, in combination with temozolomide with or without radiation therapy in patients with newly diagnosed MGMT-methylated, IDH-wild=type GBM [257]. Nowadays, according to with ClinicalTrials.gov, there are several ongoing clinical trials with regorafenib for recurrent GBM patients. For instance, the global, innovative learning environment (AGILE) is trying to evaluate multiple therapies for newly diagnosed or recurrent GBM patients [258] (Table 2).

The MIRAGE trial studies regarding the effect of regorafenib for recurrent grade 2 and 3 meningiomas are currently recruiting patients.

### 4.9. Aflibercept

The results of preclinical studies caused investigators to evaluate the responses of patients to aflibercept. Therefore, they tested the drug in clinical trials, and this led to the FDA approval of aflibercept for cancer patients. In the end, the drug was FDA approved for metastatic colon cancer in 2012 [259,260].

The first results of a phase I clinical trial of aflibercept and temozolomide in patients newly diagnosed with high-grade gliomas were published in 2011. This three-arms study evaluated the drug’s safety along with the maximum tolerated dose (MTD). The patients enrolled in arms 2 and 3 of the study were diagnosed with anaplastic glioma, including anaplastic astrocytoma, anaplastic oligodendroglioma, anaplastic mixed oligoastrocytoma or malignant astrocytoma, while in arm 1 only patients newly diagnosed with GBM were admitted. The combination was tolerated. As a result, the investigators recommended a phase II clinical trial of aflibercept with radiotherapy and concomitant temozolomide [211,261]. In the same year, the results of this phase II clinical trial were reported. As monotherapy, aflibercept had moderate toxicity and minimal benefits regarding OS in patients with recurrent GBM [212] (Table 2).

### 4.10. Ramucirumab

Ramucirumab proved to have a potential therapeutic role in solid tumors either as a single agent or in association with other chemotherapeutics [262]. As a new therapeutic option in solid tumors, the drug was tested in a series of phase I clinical trials evaluating the maximum tolerated dose as well as the toxicity [174]. All these positive results were followed by phase II and III clinical trials. Finally, in 2014, the FDA approved ramucirumab in combination with paclitaxel for advanced gastric cancer after prior chemotherapy. The RAINBOW, a double-blind randomized phase III trial, concluded that ramucirumab in combination with paclitaxel significantly prolonged OS in patients with advanced gastric cancer [263]. In the same year, the FDA expanded its approval to include treatment of aggressive non-small cell lung cancer [264]. Since then, the drug has received approval for metastatic colon cancer (as a second-line treatment in association with FOLFIRI) in 2015 [265], hepatocellular carcinoma in 2019 [266] and as a first-line treatment for metastatic EGFR-mutated non-small cell lung cancer in 2020 [267].

The preliminary results of a phase II clinical trial reported in 2013 demonstrated that the drug had a potent anti-angiogenic effect in patients with recurrent GBM [268]. This was a phase II trial which studied ramucirumab or IMC-3G3 (an anti-PDGFRα monoclonal antibody) as anti-angiogenic therapy in patients with recurrent GBM. The primary objective of the study was to assess the PFS rate at 6 months after treatment. The secondary objectives were to determine the acute and late toxicities, the tumor response rate, OS and to describe the pharmacokinetic and pharmacodynamic profiles. The patients were enrolled in two groups. Group 1 comprised recurrent GBM patients, 40 of whom received ramucirumab. The PFS at 6 months was 12.5 superior to those receiving IMC-3G3, for whom it was 7.5. The OS in the ramucirumab arm was 49.5. The data are available on ClinicalTrials.gov, ID NCT00895180: Ramucirumab or Anti-PDGFR Alpha Monoclonal Antibody IMC-3G3 in Treating Patients with Recurrent Glioblastoma Multiforme.

## 5. Conclusions

In preclinical studies and clinical trials, multi-kinase angiogenic inhibitor therapy has been established as a potential treatment for several types of cancer. Although the efficacy of multi-target angiogenic therapy has been confirmed in vitro and in vivo, it still faces several challenges concerning drug-induced adverse reactions due to toxicity; drug resistance to steady repeated therapy and poor drug biodistribution in the targeted area. In brain tumors, these angiogenic inhibitors have limited ability to cross the BBB due to their molecular size, lipophilicity and susceptibility to efflux by P-gp and BCRP. As a result, they have reduced effectiveness in treating brain tumors or brain metastases compared to other malignancies outside the brain. Researchers are continuing to investigate strategies to improve drug delivery to the brain, such as using advanced drug delivery systems, temporary BBB disruption techniques and combination therapies. Despite these difficulties, the development of more efficient angiogenic inhibitors, designed to better cross the BBB, in combination with novel administration methods to evade drug resistance, could encourage significant progress toward treating brain tumors.
